# In Vitro Biological Control of *Aspergillus flavus* by *Hanseniaspora opuntiae* L479 and *Hanseniaspora uvarum* L793, Producers of Antifungal Volatile Organic Compounds

**DOI:** 10.3390/toxins13090663

**Published:** 2021-09-17

**Authors:** Paula Tejero, Alberto Martín, Alicia Rodríguez, Ana Isabel Galván, Santiago Ruiz-Moyano, Alejandro Hernández

**Affiliations:** 1 Food Quality and Microbiology, School of Agricultural Engineering, University of Extremadura, Avda. de Adolfo Suárez, s/n, 06007 Badajoz, Spain; patejeroc@gmail.com (P.T.); amartin@unex.es (A.M.); srmsh@unex.es (S.R.-M.); ahernandez@unex.es (A.H.); 2 University Institute for Research in Agri-Food Resources (INURA), University of Extremadura, Avda. de la Investigación, s/n, 06006 Badajoz, Spain; 3 Finca La Orden-Valdesequera Research Centre (CICYTEX), Horticulture, Junta de Extremadura, 06187 Badajoz, Spain; anaisabel.galvan@juntaex.es

**Keywords:** relative gene expression, aflatoxin production, antagonism, antifungal volatile esters

## Abstract

*Aspergillus flavus* is a toxigenic fungal colonizer of fruits and cereals and may produce one of the most important mycotoxins from a food safety perspective, aflatoxins. Therefore, its growth and mycotoxin production should be effectively avoided to protect consumers’ health. Among the safe and green antifungal strategies that can be applied in the field, biocontrol is a recent and emerging strategy that needs to be explored. Yeasts are normally good biocontrol candidates to minimize mold-related hazards and their modes of action are numerous, one of them being the production of volatile organic compounds (VOCs). To this end, the influence of VOCs produced by *Hanseniaspora opuntiae* L479 and *Hanseniaspora uvarum* L793 on growth, expression of the regulatory gene of the aflatoxin pathway (*aflR)* and mycotoxin production by *A.* *flavus* for 21 days was assessed. The results showed that both yeasts, despite producing different kinds of VOCs, had a similar effect on inhibiting growth, mycotoxin biosynthetic gene expression and phenotypic toxin production overall at the mid-incubation period when their synthesis was the greatest. Based on the results, both yeast strains, *H. opuntiae* L479 and *H. uvarum* L793, are potentially suitable as a biopreservative agents for inhibiting the growth of *A. flavus* and reducing aflatoxin accumulation.

## 1. Introduction

Aflatoxins are a group of 18 highly toxic secondary metabolites produced mainly by species belonging to the genus *Aspergillus*. Among them, aflatoxins B_1_, B_2_, G_1_ and G_2_ and their metabolic products, aflatoxins M_1_ and M_2_, are the most common and threatening ones because of dietary exposure. Multiple health effects have been described related to aflatoxin exposure, such as hepatotoxicity [1], immunotoxicity [2], genotoxicity [3], changes in the microbial population of the gut [4] and reproductive damage [5]. Specifically, aflatoxin B_1_ has been classed as group I by the International Agency for Research on Cancer, which implies that this compound is carcinogenic to humans [6]. It has been reported that exposure of consumers to aflatoxin B_1_ is related to cancer in the lungs [7] and liver [8]. In order to protect consumer health, on the basis of a risk assessment, the European Commission has set maximum limits of 5–12 µg/kg for aflatoxin B_1_ and 4–15 µg/kg for the sum of aflatoxins B_1_, B_2_, G_1_ and G_2_ for oilseeds, almonds, pistachios and nuts, dried fruits, maize and cereals and spices [9].

The most worrying aflatoxin-producing species mainly belong to *Aspergillus* section *Flavi* [10]. Among the 33 species belonging to *Aspergillus* section *Flavi*, *Aspergillus flavus*, together with *Aspergillus parasiticus*, is the most concerning one. Dietary exposure to aflatoxins synthesized by this species takes place primarily because of the colonization and development of aflatoxigenic strains of vegetable-origin foods with a low water activity (a_w_) and high polysaccharide content, at both preharvest and post-harvest stages. For example, *A. flavus* has been isolated from maize-derived products [11,12], paprika [13] and dried figs [14].

Ecophysiological factors, primarily a_w_ and temperature [15], strongly affect growth and aflatoxin synthesis. Temperatures close to 30 °C and high a_w_ values (≈0.99 a_w_) encourage *A. flavus* growth, while optimal conditions for aflatoxin production are usually linked to suboptimal growth conditions [16,17,18]. The control of relative humidity and temperature during processing and storage of raw materials, ingredients and food products is an effective strategy for diminishing mold damage. However, there are other strategies that have been reported in recent years. Passone et al. [19] proposed the combination of four antioxidants to inhibit *A. flavus* development in peanuts. In addition, various essential oils, such as *Satureja hortensis* [20], turmeric, summer savory, clove [21], cinnamon [22] and *Curcuma longa* [23], present antifungal and antiaflatoxigenic properties. Furthermore, chemical fungicides, which provoke environmental and consumer health problems, are used extensively to control the growth of unwanted filamentous fungi in the field [24].

Biological control seems to be another valuable and promising strategy to control *A. flavus*. Thus, some studies have evaluated the ability of some microorganisms to control the growth of this toxigenic species, e.g., Bueno et al. [25] checked the capacity of *Lactobacillus casei* and *Lactobacillus rhamnosus* to acidify the medium, and Zhang et al. [26] tested the synthesis of antifungal compounds by *Bacillus subtillis*. Another strategy based on biological control is the selection and application of nontoxigenic strains [27,28].

In addition, the utilization of various yeast species, such as *Saccharomyces cerevisiae*, in a concentration-dependent manner [29], and *Pichia anomala,* which may inhibit spore production [30], is another effective antagonistic strategy that can be implemented against *A. flavus*.

The production of antifungal volatile organic compounds (VOCs) by biocontrol agents is one of the least explored mechanisms for the control of unwanted fungal development. Jaibangyang et al. [31] found that 49 out of 330 yeast strains were VOC producers and showed activity against *A. flavus* development. *Candida nivariensis* achieved a good level of fungal control and inhibition of aflatoxin contamination, associated with the synthesis of 1-pentanol. The compound 2-phenylethanol, the main VOC produced by *P. anomala*, has been linked to the inhibition of spore germination, growth, toxin production and gene expression of *A. flavus* [30]. In addition, it has been described that blockage or alterations in the expression of genes associated with mycotoxin biosynthesis is produced by the presence of antagonistic yeast [32]. The ubiquity of *A. flavus* and its capacity to synthesize aflatoxins in a wide range of nutritional and environmental conditions make necessary the search for efficient strategies to minimize the impact of the presence of this toxigenic mold species and mycotoxin accumulation in foods. To the best of our knowledge, there are no studies examining the production of VOCs by antagonistic yeast and their effect on aflatoxin biosynthetic genes and further phenotypic production. For this reason, it is important to identify VOCs produced by selected antagonistic yeasts in the presence of the toxigenic mold strain of interest and to investigate their influence on both mycotoxin production and the expression of a key gene involved in the aflatoxin biosynthesis pathway.

The aims of this work were to: (a) identify the VOCs produced by two yeast strains (*Hanseniaspora opuntiae* L479 and *Hanseniaspora uvarum* L793) in the presence of *A. flavus* and (b) examine the effectiveness of VOCs produced by the two antagonistic yeast strains on growth, the expression of the regulatory *aflR* gene involved in aflatoxin biosynthesis and phenotypic mycotoxin production.

## 2. Results and Discussion

### 2.1. Identification of Volatile Organic Compounds Produced by Antagonistic Yeasts

In this work, the identification of VOCs produced by the two yeasts in the presence of *A. flavus* was carried out. This was important for an in-depth analysis of whether these volatile compounds were really the main cause of reduced or minimized growth and aflatoxin production in this pathogenic fungus.

Eighty-eight different volatile compounds were identified in three batches of DDS (AF, AF + L793, AF + L479) during the 21-day incubation. The compounds identified belonged to acids (7), aldehydes (6), aromatic compounds (12), ethers (1), esters (16), furans (3), hydrocarbons (24), cyclic hydrocarbons (3), ketones (3), alcohols (8), sulphurs (1), terpenes (2) and miscellaneous compounds (2). In batches AF + L793 and AF + L479, the relative abundance of the family of volatile compounds based on total area of volatile compounds encountered was 2.87 and 1.97 times, respectively, higher than those found in the control batch (AF). The relative abundance of the family of volatile compounds was characterized by 33.38% hydrocarbons, 29.35% aromatic compounds and 15.38% alcohols in control samples without antagonistic yeasts. In the case of batch AF + L479, this was made up of 52.17% acids, 13.00% aromatic compounds, and 19.78% hydrocarbons. Finally, in the case of batch AF + L793, average values of 43.78% esters, 24.77% alcohols, 14.64% aromatic compounds and 12.25% hydrocarbons were shown. Significant differences (*p* ≤ 0.05) were found in other minority families of volatile compounds such as aldehydes, with 3.28%, 0.50% and 1.21% relative abundances for batches AF, AF + L479 and AF + L793, respectively.

Table 1 shows those VOCs that presented differences among the three batches and their mean relative abundances in each confrontation. In total, 22 compounds out of 88 presented differences among treatments. Eight of these compounds belonged to esters, four to acids, three to alcohols, two to benzene derived compounds, two to hydrocarbons, one to aldehydes, one to ketones and one to furans. In the case of batch AF + L479, *H. opuntiae* L479 produced large amounts of acetic acid (43.38%) in the presence of *A. flavus*. Other acids such as 2-methylbutanoic acid (6.91%) and isobutyric acid (1.45%) presented greater (*p* ≤ 0.05) relative abundances in batch AF + L479, whereas the alcohols, 2-methylbutanol and isoamyl alcohol decreased in relative abundances (*p* ≤ 0.001) with respect to their content in the remaining two batches (AF and AF + L793). The production of acetic acid by apiculate yeasts has been well documented [33,34], while volatile fatty acids such as 2-methylbutanoic acid and isobutyric acid have been associated with yeast metabolism during apple cider production [35]. The presence of 2-methylbutanoic acid in apple juice reduced *Penicillium expansum* growth; conversely, these compounds produced an increase in patulin accumulation [36]. Differences in the production of branched alcohols such as 2-methylbutanol and isoamyl alcohol among *Hanseniaspora* species have been proven previously [37,38].

In batch AF + L793, an increase in the abundances of esters, such as ethyl acetate (18.18%), isoamyl acetate (9.60%), 2-phenylethyl acetate (8.46%) and 2-methylbutyl acetate (3.27%), and alcohols, such as isoamyl alcohol (12.77%), 2-methyl-1-butanol (11.54%) and phenethyl alcohol (4.56%), was observed. These esters, with fruity, green and honey odors [35], have been associated with the metabolism of different *Hanseniaspora* species. For example, *Hanseniaspora osmophila* and *Hanseniaspora guilliermondii* increased the content of 2-phenylethyl acetate during wine fermentation [39,40]. Several of these esters and alcohols produced by yeasts have demonstrated antifungal properties. Ruiz-Moyano et al. [41] associated the control of *Botrytis cinerea* with the volatile esters produced by *H. uvarum* L793 cited before and other minority esters such as furfuryl acetate. *Candida maltose* produced isoamyl acetate with fungistatic properties against *Aspergillus brasilensis* [42]. *Pichia anomala*, a producer of 2-phenylethyl alcohol, has been described as an effective antagonist against *Aspergillus flavus* [30], in agreement with di Francesco et al. [43], who found that 2-pehnylethyl alcohol was the most active compound produced by *Aureobasidium pullulans* against different molds.

**Figure 1:**
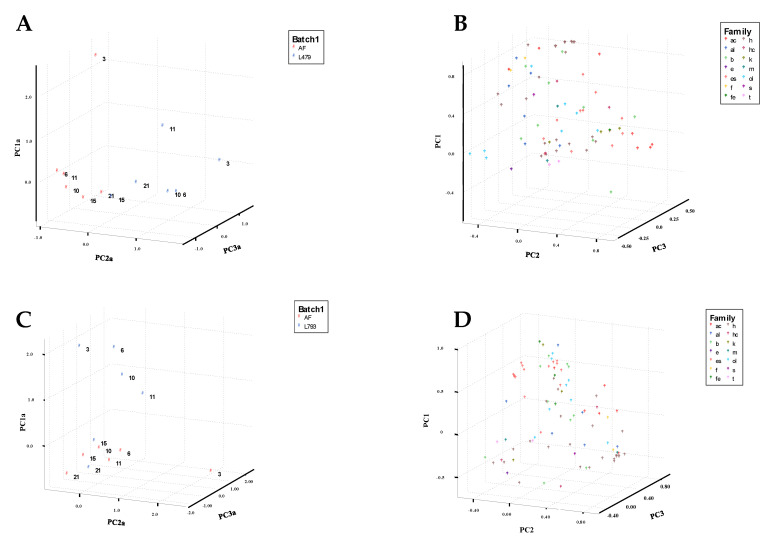
The principal component analysis (PCA) score plots (**A**,**C**) and loading plots (**B**,**D**) using the first three principal components derived from volatile compounds emitted by *A. flavus* on different sampling days (indicated by numbers), and their confrontations with *H. opuntiae* L479 (**A**,**B**) and *H. uvarum* L793 (**C**,**D**).

The composition of VOCs varied throughout the 21 days of the assay depending on the compound family. Figure 1 shows a principal component analysis (PCA) relating days of analysis to VOCs synthesized by yeasts utilized as biocontrol agents. With respect to the confrontation between *A. flavus* and *H. opuntiae* L479 (batch AF + L479; Figure 1A,B), the PCA of the three components explained 55.53% of the variability. Volatile compounds presented in batch AF (control batch), mainly composed of alcohols and hydrocarbons, were placed on the positive axis of principal component 1 (PC1) and the negative axis of PC3. The main VOCs produced by the yeast (acetic acid, 2-methylbutanoic acid and isobutyric acid) were associated with the first sampling days (3, 6 and 10 days) on the positive axis of principal component 2. Volatile compounds present on the last days of confrontations (days 15 and 21) for batches AF (control) and AF + L479 were associated with hydrocarbons and alcohols in the center of three axes.

PCA of the confrontations of *A. flavus* with *H. uvarum* L793 (batch AF + L793; Figure 1C,D) across the days of the assay explained 61.28% of the variability. On the first sampling days, this was related to esters, alcohols and aromatic compounds on the positive axes of PC1 and PC3. On the last days of incubation, compounds of AF + L793 were grouped with AF (control batch) and presented an association with hydrocarbons in the negative axes of the three principal components.

**Figure 2:**
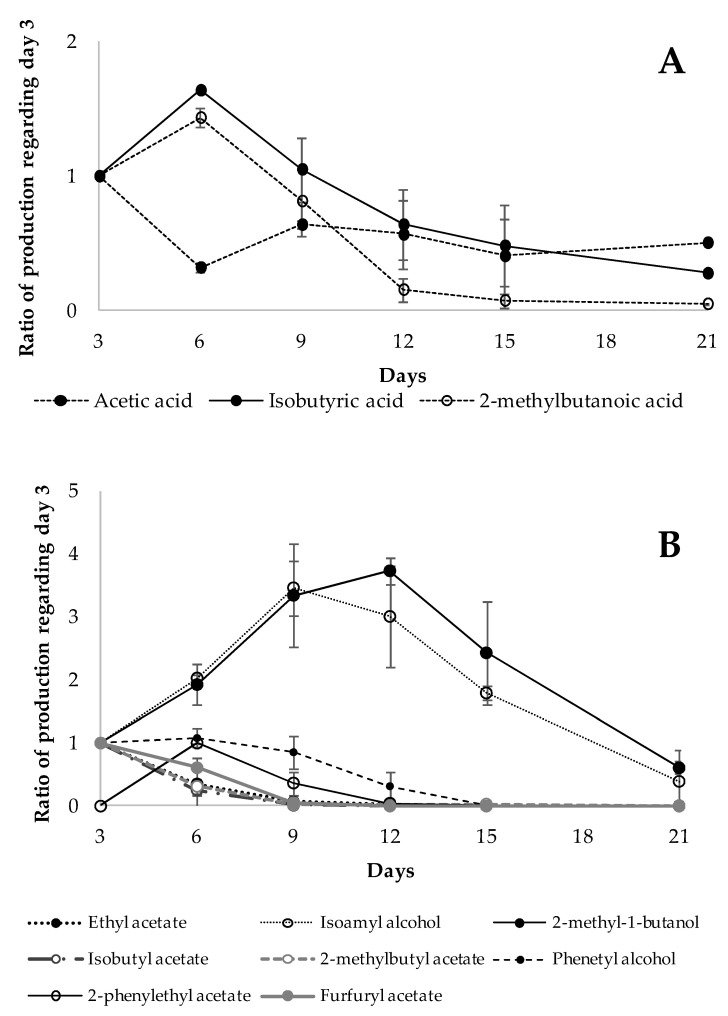
Evolution of the volatile compound profiles of *H. opuntiae* L479 (**A**) and *H. uvarum* L793 (**B**) in the presence of *A. flavus* (AF + L479 and AF + L793) throughout the 21-day incubation period.

The analysis of the evolution of the volatile profile of DDS batches showed that potential compounds involved in the control of *A. flavus* by antagonistic yeast were mainly produced in the first 12 days of the assay. Figure 2 shows the evolution of the main volatile compounds produced by the two antagonistic yeasts with respect to the initial day of analysis. Acetic acid, the most abundant volatile produced by L479, reached its maximum production at day 3, and then there was a steady decline (≈40%). After a rise in the production of the other two acids, isobutyric acid and 2-methylbutyl acid, their levels were 1.63 ± 0.00 and 1.43 ± 0.09 times higher at day 6 than at day 3, respectively; there was a noticeable decrease in values lower than the limit of detection during the remaining incubation period (Figure 2A). The main alcohols synthesized by L793, isoamyl alcohol and 2-methyl butanol, were mostly produced at days 9 and 12, respectively. Maximal production of esters was observed at 3 or 6 days of incubation (Figure 2B). Interestingly, these results disagree with the findings of Contarino et al. [44], which revealed changes in the rate of ethyl alcohol and ethyl acetate production by different antagonistic yeast species. These changes were dependent on yeast strains but, in general, ethyl alcohol was highly accumulated in the first 5 days of analysis, and ethyl acetate was the main accumulated compound after 9 days of incubation. In this study, the maximum quantities of esters as isoamyl acetate and 2-methylbutyl acetate were produced before the alcohols isoamyl alcohol and 2-methylbutyl alcohol. Previous works indicated a high capacity for ester production by *Kloeckera apiculata* (anamorph of *H. uvarum*), and hydrolyzed esters by esterases, with the possible use of acetate as a carbon source [45].

An analysis of VOCs of the two yeast-inoculated batches (AF + L479 and AF + L793) showed that both yeasts mainly synthesized such antifungal compounds during the first 12 days of the assay. However, the profiles of VOCs produced by both yeasts were different, while L479 mainly produced acetic acid, 2-methylbutanoic acid and isobutyric acid, L793 synthesized various esters, alcohols and aromatic compounds, with the main ones being 2-methyl-1-butanol and isoamyl alcohol.

### 2.2. Influence of VOCs on Growth Parameters of Aspergillus Flavus

The effect of VOCs produced by the two yeast strains tested in this study by their antagonistic activity on growth parameters of *A. flavus* was evaluated in order to analyze their capacity to inhibit or control *A. flavus* development.

Table 2 shows the size of mycelia, lag phase prior to growth and growth rate of *A. flavus* in the presence and absence of the two antagonistic yeasts (L479 and L793) during a 21-day incubation period at 25 °C. The mold in the absence of the yeasts grew from 13.55 ± 0.55 mm at day 3 to 75.20 ± 0.42 mm at day 21. A significant reduction in growth (*p* ≤ 0.05) on all sampling days was observed when *H. uvarum* L793 was coinoculated with *A. flavus*. The presence of *H. opuntiae* L479 reduced *A. flavus* growth (*p* ≤ 0.050) from day 3 to day 12 of incubation.

In the presence of L793 (batch L793 + AF), a maximum reduction in the size of *A. flavus* mycelium (34.43%) was achieved at 3 days of incubation, while a reduction of 17.10% was observed at 10 days of incubation in the presence of L479 (batch AF+ L479). Regarding the lag phase prior to growth, *A. flavus* had a shorter lag phase when inoculated alone (0.58 days) than when co-inoculated with L479 (0.87 days) and L793 (1.07 days). In the linear phase of growth, the growth rate of the control treatment was 4.58 ± 0.03 mm/day. Significant declines (*p <* 0.001) in the growth rates were observed when *A. flavus* was exposed to VOCs from antagonistic yeast strains. Growth rates of 4.00 ± 0.08 and 3.54 ± 0.08 mm/day were obtained for *A. flavus* growing in the presence of *H. opuntiae* L479 and *H. uvarum* L793, respectively.

The reduction in growth parameters of molds by VOC-producing yeasts can be attributed to inter-species differences. *Hanseniaspora opuntiae* L479 and *H. uvarum* L793 reduced both mycelial diameter and growth rate and significantly increased the lag phase of *A. flavus*. Other yeast species such as *A. pullulans*, *Filobasidium oeirense* and *Vishniacozyma carnescens* inhibited *B. cinerea* development by VOC production [41]. Jaibangyang et al. [31] found 46 out of 49 which reduced *A. flavus* mycelial growth. The high prevalence of antifungal VOC-producing strains and their high biocontrol throughout the production of volatiles could be related to the effect of CO_2_ and its synergy with VOCs [44]. *Candida nivariensis* was found to be the most effective yeast whose activity was associated with the production of alcohols such as 1-pentanol, 3-methyl-1-butanol and 2-methyl-1-propanol [31] beyond CO_2_.

It seems that the presence of VOCs can control the growth of *A. flavus*, inhibiting its mycelium diameter and lengthening the lag phase prior to growth and slowing down the growth rate of this pathogenic filamentous fungi. This effect was more pronounced when L793 was used and up to day 15 of incubation.

### 2.3. Gene Expression

**Figure 3:**
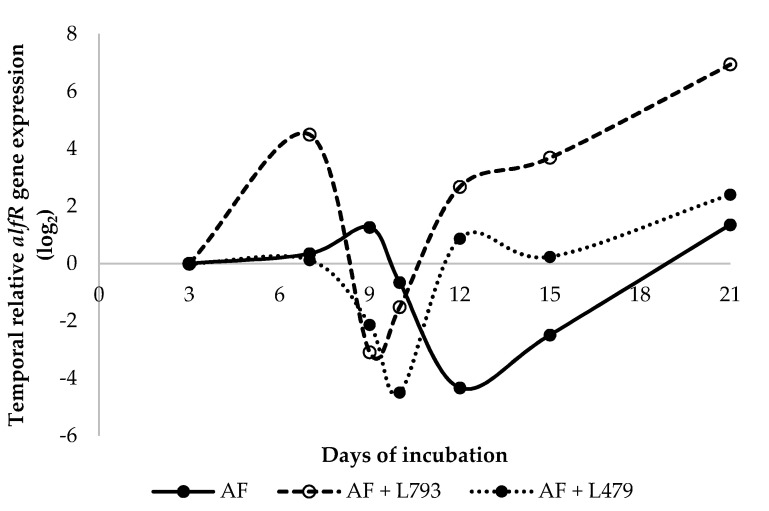
Temporal relative expression of the *aflR* gene by *A. flavus* in the absence (AF) and presence of *H. opuntiae* L479 (AF + L479) and *H. uvarum* L793 (AF + L793) throughout the 21-day incubation period. Calibrators (samples of each batch incubated at 3 days) always take the value of 0.

The effect of VOCs produced by the two antagonistic yeasts on the temporal relative *aflR* gene expression by *A. flavus* over a 21-day incubation period was evaluated (Figure 3). The *aflR* gene is a key regulatory gene involved in the aflatoxin pathway [46], and its expression has been associated with aflatoxin production under different experimental conditions [47,48]. The relative expression of the target gene at different sampling times was evaluated and compared with that in the control samples when *A. flavus* was grown in the absence (AF) and presence (AF + L479, AF + L793) of yeasts at 3 days of incubation. As can be observed in Figure 3, there were changes in the expression of the target gene throughout the incubation time. These changes agree with other studies that demonstrate that the expression of aflatoxin-related genes normally varies over time [48,49,50]. Regarding the control batch (AF), after a slow rise, where a maximum value of *aflR* gene expression was observed at day 9 of incubation, there was a steady decline in gene expression values before they increased again up until to the end of the incubation time. The results of the temporal relative *aflR* gene expression of the control batch (AF) agree with those found in previous studies. Schmidt-Heydt et al. [51,52,53] suggested that the expression of aflatoxin-related genes was optimal after 8–10 days of growth. Regarding both yeast- and *A. flavus*-inoculated batches (AF + L479 and AF + L793), in general, their *aflR* gene expression patterns were quite similar (Figure 3), with only small differences. In the case of batch AF + L479, *aflR* gene expression values remained constant during the first 6 days of incubation before decreasing noticeably at days 9–10 of incubation and then increasing considerably at day 12. Later, after a slight decrease, there was a gene expression activation on day 21. In the case of batch AF + L793, there was a dramatic rise in *aflR* gene expression on day 6 of incubation. After that, substantial inhibition of expression of this gene was observed on day 9 of incubation. Next, *aflR* gene expression values generally increased at each incubation time to reach their highest levels at the end of the incubation period (21 days). Results from both batches inoculated with yeast producers of VOCs cannot be compared with those of previous studies, since this is the first time that temporal relative gene expression profiles have been carried out in order to determine the influence of these antifungal compounds on the expression of a mycotoxin–gene biosynthetic pathway. It is important to highlight that, at day 9 of incubation, VOCs produced by the yeast strains L793 and L479, respectively, could inactivate *aflR* gene expression only when the maximum expression of this gene was found in the control batch (AF), in accordance with findings published by Schmidt-Heydt et al. [51], who demonstrated that the expression of the *aflR* gene reached its maximum at 9–10 days of incubation. In addition, it is also significant to point out that the *aflR* gene expression values of batches AF + L479 and AF + L793 were higher than those encountered in the control batch (AF) at the final sampling time (21 days).

**Figure 4:**
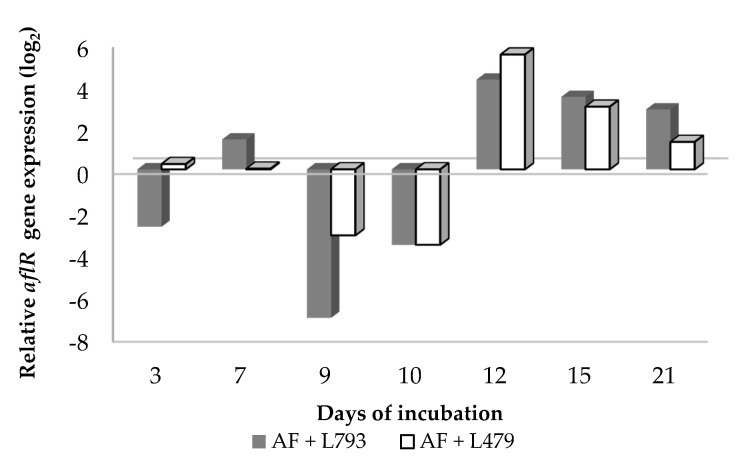
Effect of volatile organic compounds produced by *H. opuntiae* L479 (AF + L479) and *H. uvarum* L793 (AF + L793) on the relative expression of the *aflR* gene in *Aspergillus flavus* throughout the 21-day incubation period. Calibrators (samples of batch AF at each incubation time) always take the value of 0.

Figure 4 shows the expression of the *aflR* gene by *A. flavus* when confronted with VOCs produced by yeasts L479 and L793 at each incubation time. The results show that there was an inhibition of the expression of the aflatoxin regulatory gene at days 9 and 10 of incubation in the presence of VOCs synthesized by the two yeasts, as well as at the first sampling day (3 days) in the case of L793. Moreover, activation of the expression of the aflatoxin-related gene was observed from day 12 of incubation until the end of the incubation period. These results coincided with previous studies demonstrating that a biocontrol agent does not always provoke inhibition of the expression of a mycotoxin-biosynthetic gene [47,54]. The presence of antifungal compounds may activate such mycotoxin biosynthetic routes.

### 2.4. Aflatoxin Amounts

**Figure 5:**
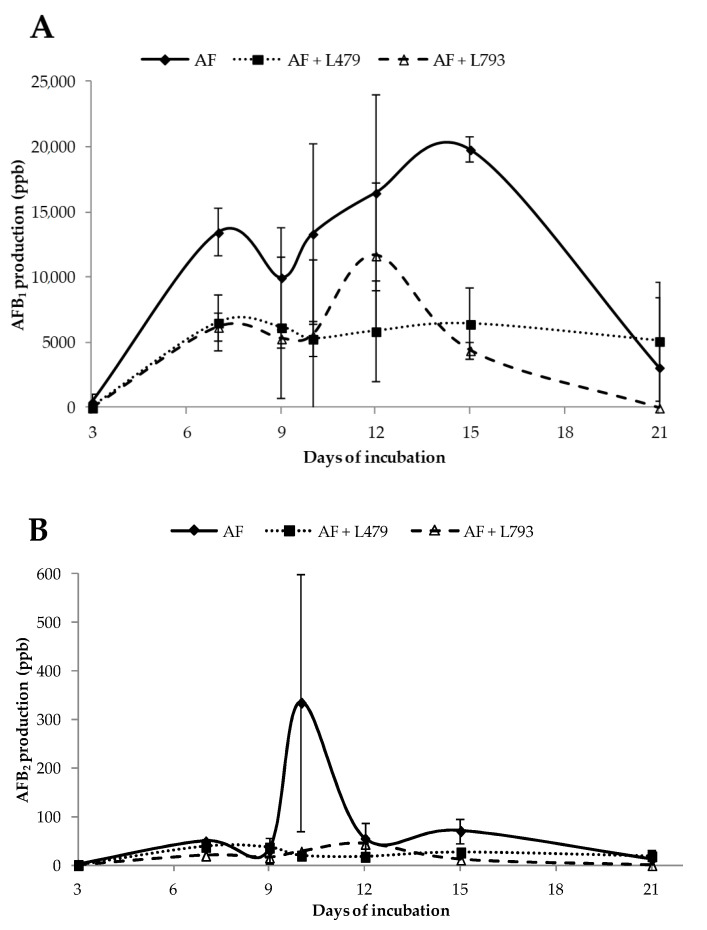
Temporal aflatoxin B_1_ (**A**) and B_2_ (**B**) production by *A. flavus* in the absence (AF) and presence of *H. opuntiae* L479 (AF + L479) and *H. uvarum* L793 (AF + L793) throughout the 21-day incubation period.

Figure 5 shows the effect of VOCs produced by *H. opuntiae* L479 and *H. uvarum* L793 on AFB_1_ and AFB_2_ production by *A. flavus* during a 21-day incubation period. Both aflatoxins were inhibited in the presence of VOCs produced by both yeasts (*p <* 0.001 and *p* = 0.003, respectively). However, AFB_1_ amounts were significantly higher than AFB_2_ quantities produced by the aflatoxigenic strain in the presence and absence of both yeast strains (*p* ≤ 0.050). These results agree with those previously published by Schmidt-Heydt et al. [51] and Galván et al. [18].

Regarding AFB_1_, the amount produced by *A. flavus* in the control batch on the first sampling day (day 3) was 451 ± 621 ppb. The amount of toxin increased considerably from day 3 to day 15 (19,813 ± 981 ppb) and then fell substantially in the final days of incubation (3102 ± 5372 ppb). The quantities of AFB_1_ in the presence of antagonistic yeasts began with 47 ± 11 and 3 ± 3 ppb for the AF + L479 and AF + L793 batches, respectively. The presence of AFB_1_ remained stable in the AF + L479 confrontations from day 7 to day 21, with concentrations around 5000–6000 ppb. A peak of AFB_1_ production was observed at 12 days in the AF + L793 batch, with values of 11,669 ± 5554 ppb. Finally, no AFB_1_ was detected at 21 days of incubation in this batch. Generally, AFB_1_ amounts were inhibited by the action of VOCs produced by both yeasts at each incubation time. From day 3 to day 10 of incubation, both yeasts had a similar effect on toxin synthesis; however, on day 12 of incubation, L479 provoked a higher reduction in toxin amounts than L793, while on the last three incubation days tested, the last yeast strain had a greater influence on minimizing the amount of AFB_1_ synthesized by *A. flavus.* These findings coincide with those reported by Al-Saad et al. [47] and Peromingo et al. [54], who used biocontrol agents to control the carcinogenic toxin amounts produced by this filamentous fungal species. Thus far, no studies have been conducted to evaluate the impact of VOCs synthesized by microorganisms on mycotoxin production.

In terms of AFB_2_ production, the amounts of this toxin varied between 0.9 and 334 ppb in the control batch, but this increase was not constant throughout the incubation time: a main peak of production was observed at day 10 of incubation and a secondary peak at day 15 of incubation. In the case of batches AF + L479 and AF + L793, *A. flavus* produced similar quantities of this mycotoxin that practically levelled off throughout the incubation time. The main difference found between the two abovementioned batches was that, in the presence of strain L479, *A. flavus* synthesized AFB_2_ at the end of the incubation period, whereas, in the presence of strain L793, it did not. In this case, the greatest inhibition of production of this toxin by VOCs produced by both yeasts was encountered at day 10 of incubation.

## 3. Conclusions

This study proved that the two antagonistic yeasts evaluated in this work (*H. opuntiae* L479 and *H. uvarum* L793) mainly produced VOCs in the presence of *A. flavus* during the first 12 days of incubation. Although the VOCs synthesized by the two yeast strains were chemically different, their influence on *A. flavus* development and colonization was quite similar. *H. uvarum* L793 strictly controlled this toxigenic filamentous fungus throughout the entire incubation period; however, the two yeasts had a similar inhibitory effect on the expression of the aflatoxin regulatory gene *aflR* and mycotoxin production overall at the midpoint of the incubation period (9–10 days) when the synthesis of VOCs by yeasts reached its maximum. Based on the results, both yeast strains, *H. opuntiae* L479 and *H. uvarum* L793, are potentially suitable as biopreservative agents for inhibiting the growth of *A. flavus* and reducing aflatoxin accumulation. It seems that the application of both antagonistic yeast strains at the early post-harvest stages of fruit or cereals is critical to minimize health hazards due to aflatoxin development in these types of products. In further studies, inoculation of *H. opuntiae* L479 and *H. uvarum* L793 several times during the post-harvest handling of these vegetable-origin foods should be evaluated. 

## 4. Materials and Methods

### 4.1. VOC-Producing Yeasts

Two different strains representing different species of antifungal-VOC-producing yeast were used in this work. *H. opuntiae* L479 and *H. uvarum* L793 were previously selected for their capacity to produce antifungal VOCs [41]. Cryogenized yeast strains were inoculated on acidified (10% tartaric acid, Sharlab, Barcelona, Spain) potato dextrose agar (PDA, Scharlab) and incubated at 25 °C for 48 h. A loop of the fresh yeast culture was recovered and resuspended in sterile distilled water. Cells were counted using a Neubauer chamber, adjusted to a final concentration of 2 × 10^7^ cells/mL and used as inocula. Both yeast strains can be ceded to other research groups for scientific purposes by a material transfer agreement between both research groups.

### 4.2. Aspergillus Flavus Strain

The *A. flavus* CQ8 strain was taken from the Fungi Culture Collection of the Agricultural Engineering School (Extremadura University, Badajoz, Spain) and was previously characterized by Casquete et al. [13]. Cryogenized conidia were seeded on acidified PDA and cultivated at 25 °C until complete sporulation. Conidia were recovered with 0.05% of Tween 80 solution (*v*/*v*, Sigma-Aldrich, Madrid, Spain) and quantified by direct observation in an optical microscope with a Neubauer chamber. Conidia solution was diluted up to 10^6^ conidia/mL and used as inoculum.

### 4.3. Competitiveness of VOC-Producing Yeasts against Aspergillus Flavus Assessed Using Double-Dish Systems

The antagonistic yeast strains (*H. uvarum* L793 and *H. opuntiae* L479) and *A. flavus* were confronted in double-dish systems (DDS) essentially as described by Ruiz-Moyano et al. [41]. Briefly, 5 µL of *A. flavus* inoculum was inoculated onto the center of the bottom PDA plate, which had previously been covered by a porous cellophane disc (Packaging Limited, UK). Then, 100 µL of each of the two antagonistic yeast solutions was inoculated separately with a Digralsky spatula on the upper plate. Subsequently, both Petri plates were joined with a piece of Parafilm^TM^. Finally, four holes (3 mm × 5 mm) were perforated in the union of both plates. A control set of DDS was performed without the presence of antagonistic yeasts. The study was composed of three DDS batches: (a) AF + L793 (*A. flavus* in the presence of *H. uvarum* L793), (b) AF + L479 (*A. flavus* in the presence of *H. opuntiae* L479) and (c) AF (*A. flavus* in the absence of yeasts, control batch). The three batches were stored at 25 °C, and sampling was carried out at 3, 7, 9, 10, 11, 12, 15 and 21 days of incubation. Growth parameters, *aflR* gene expression and aflatoxin production were determined on each sampling day. The assay was conducted twice, and three replicates were performed for each repetition. 

### 4.4. Analysis of Volatile Compounds

Extraction and analysis of VOCs produced by the two yeast strains in the presence and absence of the filamentous fungus were conducted as described by Ruiz-Moyano et al. [41]. These volatile compounds were extracted by using a 10-mm long, 75-μm thick fiber coated with carboxen/polydimethylsiloxane from the space of each DDS by solid-phase microextraction (SPME) (Supelco, Bellefonte, PA, USA). The origin of volatile compounds from PDA and *A. flavus* was assigned by extraction and analyses of batch AF.

After volatile compound extraction, analyses were conducted by gas chromatography mass spectrometry (GC/MS) using an Agilent 6890 GC-5973 MS system (Agilent Technologies, Little Falls, DE, USA) equipped with a 5% phenyl-95% polydimethylsiloxane column (30 m × 0.32 mm inner diameter, 1.05 μm film thickness, Hewlett-Packard). The Kovats index of the compounds was calculated by analysis of n-alkanes (R-8769, Sigma Chemical Co., St. Louis, MO, USA) run under the same conditions as the samples. The NIST/EPA/NIH mass spectrum library (comparison quality > 90%) and Kovats index were used to identify the volatile compounds produced by the two yeast strains. Additionally, the identity of certain compounds was confirmed by a comparison of the retention time and MS spectra, using a laboratory-built MS spectral database, obtained from chromatographic runs of pure compounds performed under the same experimental conditions by using the same equipment. Quantitative data were obtained from the total ion current chromatograms by integration of the GC peak areas.

The volatile compounds associated with yeast strains in batches AF + L479 and AF + L793 were determined by comparison of volatile compounds found in such batches with those encountered in a PDA control without yeast inocula and in batch AF (batch control inoculated only with *A. flavus*). The production of those volatile compounds that were not detected in both control PDA and PDA inoculated with *A. flavus* (batch AF), or those whose relative abundances were significantly lower than those encountered in yeast-inoculated batches (AF + L479 and AF + L793), was exclusively linked to the strains *H. opuntiae* L479 and *H. uvarum* L793 according to the methodology described in Ruiz-Moyano et al. [41].

### 4.5. Determination of Growth Parameters of Aspergillus Flavus

The diameter of the *A. flavus* colony was measured in two perpendicular directions and recorded on each sampling day. Growth curves were obtained by graphical representation of the mycelium diameter (mm) against the incubation times (days). Data plots showed, after a lag phase, a linear trend with time; therefore, a linear model was applied. The growth rate (µ; mm/day) was determined from the slope of the growth curve during the linear phase of growth. The lag phase (λ; days) was determined from the linear regression equation equaling the regression line formula to the original inoculum size (diameter, mm) according to León et al. [55].

### 4.6. Relative Quantification of the Expression of the aflR Gene

#### 4.6.1. Sample Preparation

After each incubation period, the mycelium was scraped from the surface and collected under sterile conditions, quickly frozen in liquid nitrogen and stored at −80 °C until RNA extraction.

#### 4.6.2. RNA Extraction

Frozen mycelium was used for RNA extraction with the Spectrum^TM^ Plant Total RNA Kit (Sigma-Aldrich). RNA concentration (µg/mL) and purity (A260/A280 ratio) were determined using a 1.5-μL aliquot on a NanoDrop^TM^ spectrophotometer (Thermo Fisher Scientific, Madrid, Spain). Samples were diluted to 0.1 μg/μL and treated with DNAse I (Thermo Fisher Scientific) to remove genomic DNA traces that could be co-extracted with RNA.

#### 4.6.3. Two-Step Reverse-Transcription Real-Time PCR

##### Retrotranscription Reaction

Synthesis of complementary DNA (cDNA) was carried out using 5 µL of total RNA according to the manufacturer’s instructions of the PrimeScript™ RT reagent Kit (Takara Bio Inc., Kusatsu, Shiga, Japan). The reaction conditions were incubation at 37 °C for 15 min and reverse transcriptase inactivation at 85 °C for 5 s. Then, cDNA samples were stored at −20 °C until gene expression analysis.

##### Real-Time PCR Reactions

The real-time PCR (qPCR) reactions were conducted in a 7300 Real-Time PCR System (Applied Biosystems, Carlsbad, CA, USA) using SYBR^®^ Green technology. The amplification of *aflR* and *β-tubulin* genes was conducted according to the methodology described by Peromingo et al. [48].

Briefly, the final volume of the reaction mixture for the amplification of each gene was 12.5 μL and consisted of 6.25 μL of SYBR^®^ Premix Ex Taq™ (Takara Bio Inc., Kusatsu, Japan), 0.05μL of ROX plus (Takara Bio Inc.) and 2.5μL of cDNA template. For the *aflR* gene, the final concentration of the primer pair AflRTaq1/AflRTaq2 was 300 nM each, while that of the primers F-TUBjd/R-TUBjd used to amplify the *β-tubulin* gene was 400 nM each.

The thermal cycling conditions for amplification of both genes included one initial denaturation step at 95 °C for 10 min, and 40 cycles at 95 °C for 15 s and 60 °C for 30 s. After the final PCR cycle, melting curve analyses of the PCR products were conducted and checked to ensure the fidelity of the results.

The quantification cycle (Cq), the cycle in which fluorescence reaches a defined threshold, was automatically calculated by the instrument using the default parameters of the 7300 Fast System Software (Applied Biosystems).

#### 4.6.4. Calculation of Relative Gene Expression

Relative quantification of the expression of the *aflR* gene was basically performed as previously detailed by Peromingo et al. [48]. The expression ratio was calculated using the 2^−ΔΔCT^ method [56]. The *β-tubulin* gene was used as an endogenous control. Calibrators corresponded to the *A. flavus* strain grown in the absence of yeast (batch AF, control), and the samples were incubated for 3 days (first sampling day).

### 4.7. Aflatoxin Analysis

Aflatoxin extraction was conducted per the method described by Ruiz-Moyano et al. [57], with some modifications. The content of one Petri dish was transferred to a filter plastic bag and macerated with 100 mL of chloroform in a Stomacher Lab-Blender 400 (Seward Medical, Worthing, UK) for 2 min. After 1 h in darkness at room temperature, the slurry was filtered twice through anhydrous sodium sulphate with Whatman no. 1 filter paper (Whatman International, Maidstone, UK). Then, the filtrate was evaporated in a rotatory evaporator model Hei-Vap (Heidolph, Schwabach, Germany) at 37 °C. The residue was resuspended in 6 mL of chloroform, transferred to a new tube and evaporated to dryness in a Myvac vacuum centrifuge evaporator (Genevac, Winchester, UK) heated to 37 °C. The residue was resuspended in 1 mL of HPLC-grade methanol, followed by the addition of 1 mL of HPLC-grade water and filtered through a 0.22-μM pore-size filter into HPLC vials for quantification.

Aflatoxin concentration was determined using an Agilent 1100 Series HPLC system (Agilent Technologies, USA) coupled to a fluorescence detector (Agilent) using a SUPELCOSIL LC-18 column (15 cm × 4.6 mm, 5-µm particle size; Supelco, Bellefonte, PA, USA) after post-column derivatization with pyridinium bromide at 0.005% (*w*/*v*; Sigma). The post-column derivatization reagents were pumped at 0.3 mL/min by means of an HPLC pump from an Agilent 1100 Series HPLC apparatus. The fluorescence detector was set to excitation and emission wavelengths of 360 and 430 nm, respectively. Separation of aflatoxins was achieved with a mobile phase containing a mixture of MeOH:ACN:water (20:20:60 *v*/*v*/*v*), which was delivered at an isocratic flow rate of 1 mL/min. All solvents used were of HPLC grade (Thermo Fisher Scientific). The injection volume was 100 μL and calibrations were carried out with an aflatoxin mix standard (AFB1, AFB2, AFG1 and AFG2) purchased from Sigma-Aldrich.

### 4.8. Statistical Analysis of Results

Data for growth parameters, aflatoxin production, relative gene expression and volatile compound areas at each sampling day of confrontations were analyzed separately using IBM SPSS Statistics, Version 19.0. (Armonk, NY, USA: IBM Corp.). Differences in the mean values of parameters were tested by one-way analysis of variance (ANOVA) followed by Tukey’s honestly significant difference test (*p* ≤ 0.050). In addition, PCA was performed to relate the days of analysis to the family of VOCs synthesized by the yeasts (*H. opuntiae* L479 and *H. uvarum* L793) utilized as biocontrol agents in this study to control *A. flavus*.

## Figures and Tables

**Table 1:** Volatile compounds (including family, Kovats index and retention times) and their relative abundances in the three batches analyzed in this study: AF (batch inoculated only with *Aspergillus flavus*), AF + L479 (batch inoculated with *Aspergillus flavus* and *Hanseniaspora opuntiae* L479) and AF + L793 (batch inoculated with *Aspergillus flavus* and *Hanseniaspora uvarum* L793).

Volatile Compounds	Family ^a^	KI ^b^	Peak Number	Retention Time	Mean Relative Abundances			*p*
					AF ^c^	AF + L479 ^d^	AF + L793 ^e^	
1-Propanol	ol	554	v4	5.1	0.28	0.00 *	0.08 *	0.032
2-Methylpentane	h	560	v5	5.3	2.29	0.73 *	0.23 *	0.024
3-Methylpentane	h	581	v6	5.8	2.58	0.95 *	0.25 *	0.050
Acetic acid	ac	595	v9	6.8	0.00	43.38 +	0.00	0.000
Ethyl acetate	es	612	v10	6.8	1.89	2.59 +	18.184 +	0.023
Propanoic acid	ac	705	v20	10.7	0.09	0.22 +	0.24 +	0.008
n-Propyl acetate	es	706	v22	11.0	0.00	0.35 +	0.70 +	0.049
Isoamyl alcohol	ol	732	v26	11.9	9.54	0.81 *	12.77 +	0.001
2-Methyl-1-butanol	ol	740	v27	12.1	4.36	0.43 *	11.54 +	0.000
Isobutyric acid	ac	775	v29	12.8	0.84	1.45 +	0.41	0.021
2-Methylbutanoic acid	ac	854	v42	16.8	0.59	6.91 +	0.28	0.017
1,3-Dimethylbenzene	b	875	v44	18.8	0.72	0.31 *	0.10 *	0.047
Isoamyl acetate	es	878	v45	18.9	0.33	0.32	9.60 +	0.043
2-methylbutyl acetate	es	880	v46	19.0	0.61	0.30	3.27 +	0.039
2-Heptanone	k	889	v48	19.5	0.00	0.03 +	0.30 +	0.007
Furfuryl acetate	f	997	v57	24.4	0.00	0.00	0.26 +	0.006
Hexyl acetate	es	1010	v58	25.1	0.00	0.03 +	0.13 +	0.048
Ethyl heptanoate	es	1085	v67	28.7	0.00	0.03 +	0.08 +	0.048
Nonanal	al	1104	v68	29.1	0.20	0.08 *	0.23 +	0.046
Phenethyl alcohol	b	1110/1141	v69	29.8	0.00	0.00	4.56 +	0.007
Phenylmethyl acetate	es	1170	v70	31.6	0.00	0.00	0.13 +	0.043
2-Phenylethyl acetate	es	1258	v76	35.0	3.02	1.48	8.46 +	0.045

^a^ ol: alcohols; h: hydrocarbons; ac: acids; es: esters; b: benzene derivates; k: ketones; al: aldehydes; f: furans; al: aldehydes. ^b^ Kovats index. Batches: ^c^ AF (*A. flavus* in the absence of yeasts, control batch), ^d^ AF + L793 (*A. flavus* in the presence of *H. uvarum* L793), ^e^ AF + L479 (*A. flavus* in the presence of *H. opuntiae* L479). * Means with lower relative abundances (*p <* 0.050). + Means with higher relative abundances (*p <* 0.050).

**Table 2:** Growth parameters (size of mycelia), growth rate (µ; mm/day) and lag phase (λ; days) of *Aspergillus flavus* in the absence (AF) or presence of *H. opuntiae* L479 (AF + L479) or *H. uvarum* L793 (AF + L793).

Treatment	Diameter of Mycelium (mm)							µ (mm/Day)	λ (Days)
	Days of Incubation								
	3	7	9	10	12	15	21		
AF	13.55 ± 0.52c ^1^	34.50 ± 1.11c	43.72 ± 0.75b	47.50 ± 0.74c	57.55 ± 1.83c	70.83 ± 0.96b	75.20 ± 0.44b	4.58 ± 0.03c	0.58 ± 0.04a
AF + L479	12.00 ± 0.50b	29.74 ± 0.97b	37.95 ± 1.84a	39.37 ± 0.99b	50.26 ± 4.18b	63.87 ± 4.38b	73.20 ± 2.38b	4.00 ± 0.08b	0.87 ± 0.10b
AF + L793	8.88 ± 1.26a	25.39 ± 1.93a	32.36 ± 2.60a	35.55 ± 2.85a	42.81 ± 3.47a	52.00 ± 5.13a	57.00 ± 7.37a	3.54 ± 0.08a	1.07 ± 0.08b
*p*	<0.001	<0.001	<0.001	<0.001	<0.001	<0.001	0.015	<0.001	0.001

Data are expressed as mean value ± standard deviation. ^1^ within columns, different letters denote significant differences for the same incubation day between treatments (*p* ≤ 0.05).

## Data Availability

The data presented in this study are available in Tejero, P.; Martín, A.; Rodríguez, A.; Galván, A.I.; Ruíz-Moyano, S.; Hernandez, A. In Vitro Biological Control of *Aspergillus flavus* by *Hanseniaspora opuntiae* L479 and *Hanseniaspora uvarum* L793, Producers of Antifungal Volatile Organic Compounds. Toxins 2021, 13, 663, doi:10.3390/toxins13090663.
